# *Curvularia lunata* drives biodeterioration of PVC secondary cable insulation involving surface colonization, moisture retention and chemical deterioration

**DOI:** 10.1371/journal.pone.0357774

**Published:** 2026-09-08

**Authors:** Yimeng Liu, Shiying Yan, Jian Zhang, Yiping Chen, Yue Zhou, Changyi Huang, Tianlan Jiang, Yu Gao, Hansheng Zhu, Hao Shi, Chaoyi Han, Fosheng Li, Jie Zhang, Jian Zhao, Mei Cao

**Affiliations:** 1 Key Laboratory of Biological Resource and Ecological Environment of Chinese Education Ministry, College of Life Sciences, Sichuan University, Chengdu, Sichuan, China; 2 Animal Disease Prevention and Green Development Key Laboratory of Sichuan Province, College of Life Sciences, Sichuan University, Chengdu, Sichuan, China; 3 Core Laboratory, Sichuan Provincial People’s Hospital, School of Medicine, University of Electronic Science and Technology of China, Chengdu, Sichuan, China; National Research and Innovation Agency, INDONESIA

## Abstract

Microbial degradation of cable insulation materials is a critical issue affecting the reliability of power systems. In this study, metagenomic analysis was employed to reveal the microbial community structure on contaminated substation cables, identifying *Curvularia lunata* as a dominant fungal species in high-voltage environments. Subsequently, a specific strain, *Curvularia lunata B3*, was isolated and identified for further investigation. To assess its specific impact on insulation performance, artificial inoculation experiments were conducted on secondary cable samples. Multi-dimensional characterization techniques, including SEM, WCA measurements, halogen moisture analysis, FTIR, XPS, and LCR digital bridge testing, were utilized to evaluate material degradation. The results demonstrated that *C. lunata* colonization caused significant surface erosion, characterized by the formation of holes and furrows. This physical damage was accompanied by a marked decrease in hydrophobicity, with the water contact angle dropping from 88.30 ± 0.79° to 78.27 ± 1.27°, and a gradual increase in water content to approximately 1.2% over 60 days. Chemical analysis revealed that microbial activity induced oxidation and dechlorination of the PVC insulation, evidenced by the reduction of C-Cl bonds and the emergence of oxygen-containing functional groups. These physicochemical alterations significantly compromised the electrical insulation of the cables, as evidenced by a marked decrease in series resistance (Rs) and an increase in series capacitance (Cs). This study elucidates the mechanisms of fungal erosion on cable insulation and provides a scientific basis for developing targeted protective strategies in power systems.

## Introduction

Ensuring the reliable operation of power systems is crucial for modern society. Substations, as pivotal nodes, rely heavily on secondary cables for signal transmission and control. These cables are frequently situated in challenging environments characterized by high temperatures and humidity, making them prone to the accumulation of dust, moisture, and diverse microbial communities. The proliferation of these microorganisms poses a considerable risk to the durability and efficacy of cable insulation [1]. Microbial activities can degrade insulation performance, potentially leading to electrical faults and large-scale blackouts, thereby compromising the safety and reliability of the power grid [2].

While material degradation in power systems is a recognized issue, the precise impact of microbial contamination on secondary cable insulation, including the intricate mechanisms underlying this deterioration, remains inadequately elucidate [3]. Conventional microbial analysis methods often offer limited insights into the intricate microbial communities at play, failing to fully capture their diversity and potential impact on erosion [4]. Moreover, there is a pressing need to comprehensively and multidimensionally characterize how distinct functional microorganisms influence the physicochemical and electrical properties of cable insulation materials; however, research in this domain remains inadequate [3]. While some studies have investigated microbial erosion of polymeric materials, a systematic approach that integrates microbial community analysis with detailed material characterization techniques to establish correlations between specific microbial activities and resultant changes in cable performance within an operational substation environment is still nascent [5].

To address these knowledge gaps, a comprehensive investigation was conducted to assess the impact of microbial contamination on secondary cable insulation. Initially, metagenomic sequencing was employed to analyze the microbial community structure on contaminated substation cables and identify predominant species. This advanced method enhanced the comprehension of microbial diversity at the phylum and genus levels, pinpointing species associated with specific environmental conditions, such as varying voltage levels. Notably, *Curvularia lunata* emerged as a significant fungal species in high-voltage environments. Following the isolation and characterization of *Curvularia lunata B3*, controlled artificial inoculation experiments were performed on cable samples to simulate the erosion process. The impact of *Curvularia lunata* on cable material properties was rigorously assessed employing diverse characterization techniques, including scanning electron microscopy (SEM), water contact angle (WCA) analysis, halogen moisture content measurements, Fourier transform infrared spectroscopy (FTIR), X-ray photoelectron spectroscopy (XPS), and LCR digital bridge measurements [3,6–9].

This study aims to systematically investigate the role of distinct microbial activities in the degradation of secondary cable insulation. Through the integration of metagenomic analysis and thorough material science characterization, we seek to uncover the fundamental mechanisms of microbial degradation and its consequential effects on the electrical functionality of power cables. The outcomes of this research are anticipated to establish a theoretical framework and offer practical recommendations for developing efficient strategies to mitigate microbial degradation, thereby enhancing the operational reliability and longevity of secondary cables within substations.

## Materials and methods

### Metagenomic analysis

To analyze the metagenomic composition of microbial contaminants on the cable, we conducted the following procedures: First, microbial samples were collected from the contaminated cable surfaces of 20 cables within each voltage class group, and genomic DNA was extracted using a commercial DNeasy PowerSoil Pro Kit to ensure the integrity and purity of the DNA. Subsequently, metagenomic sequencing libraries were constructed using the NEBNext Ultra DNA Library Prep Kit for Illumina, and sequencing was performed on an Illumina NovaSeq 6000 platform with 2x150 bp paired-end reads to obtain high-quality raw data. In the bioinformatics analysis phase, we first used fastp software (version 0.23.2) for stringent quality control of the raw data, which included adapter trimming and filtering out low-quality reads. To ensure accuracy in downstream quantification, duplicate reads were systematically identified and removed. The clean reads were then normalized across all samples (calculated as relative abundance) to eliminate sequencing depth bias. The cleaned data were then aligned against the NCBI NT database using Kraken2 software for taxonomic annotation. Finally, we calculated the relative abundance of microbial communities at different taxonomic levels (e.g., phylum, genus) and used the ggplot2 package in R to generate the taxonomic composition plots, thereby systematically analyzing the characteristics of the contaminated microbial community.

### Morphological characterization of *Curvularia lunata*

The fungal strain used in this study, identified as *Curvularia lunata B3*, was isolated from a contaminated cable sample. To characterize its morphology, we conducted observations at both macroscopic and microscopic levels. For macroscopic analysis, the strain was inoculated onto potato dextrose agar (PDA) plates and incubated at 28℃ in the dark for 7 days. After incubation, a digital camera was used to document the macroscopic morphological features of the colony, including its color, texture, and growth pattern. For microscopic analysis, we used a slide culture method for live-cell observation. Briefly, a drop of unsolidified PDA medium was placed on a sterile glass slide and inoculated with fungal spores. The prepared slide was then incubated in a Petri dish at 28℃ for 3–4 days. Once hyphae had grown, the sample was directly observed and photographed under a digital camera-equipped optical microscope (Olympus DP73, Japan) without any staining. This systematic documentation of the morphology of both hyphae and conidia, including their size, shape, septation, and arrangement, was used to provide microscopic evidence for the species identification of the strain.

### Artificial inoculation and erosion experiment

For this study, we prepared cable samples using ZA-KVVP2–22 4 × 4 type (450/750 V) secondary cables, with each sample measuring 50 cm in length. First, the cable samples were coiled into a ring with an approximate diameter of 6 cm and secured with plastic ties before being placed in a 2000 mL beaker. The samples were then immersed in a 75% ethanol solution for 6 hours for disinfection. Following this, they were transferred to a sterile workbench for 30 minutes of UV irradiation to ensure complete sterilization, then moved to a new sterile beaker for subsequent use. The control group samples underwent the exact same disinfection and sterilization procedures.

Before inoculation, the *Curvularia lunata B3* spore solution, stored at −40℃ with 15% glycerol as a cryoprotectant, was activated in potato dextrose broth (PDB) at 28℃ and 120 rpm for 48 hours. Simultaneously, a potato dextrose agar (PDA) medium with a 4% agar concentration was prepared. After the medium cooled to a suitable temperature, the activated fungal mycelial pellets were added, mixed thoroughly, and poured evenly into the beaker containing the cable samples. Finally, the beaker was sealed with plastic wrap to create an isolated environment, and the samples were incubated in a 28℃ biological incubator. All subsequent characterization tests in this study were performed on contaminated cable samples prepared using this method.

### Scanning electron microscopy (SEM) characterization

For this study, a scanning electron microscope (SEM) was used to characterize the surface morphology and microstructure of the cable samples. The instrument used was an Apreo S SEM (Thermo Fisher Scientific, USA). Samples were selected from an uncontaminated control group as well as from cables with contamination periods of 3, 6, 9, and 12 months.

Prior to SEM analysis, all samples were pretreated to remove surface impurities. First, the samples were placed in an ultrasonic cleaner at 30℃ for 10 minutes. This was followed by a gentle brushing with a soft brush to dislodge any residual fungal biomass and culture medium before being placed back in the ultrasonic cleaner for a second 10-minute cleaning. After cleaning, the cable samples were cut into small square pieces (approximately 3 mm × 3 mm) using scissors and tweezers disinfected with 75% ethanol. These pieces were then securely attached to a conductive sample stub. Finally, to enhance conductivity and improve imaging quality, all samples were pretreated with gold sputter-coating for 150 seconds before testing.

### Contact angle analysis

For this study, we performed a static contact angle test to characterize the surface wettability of cable samples before and after contamination, which in turn allowed us to analyze changes in their surface energy. The test was conducted using a DSA100 contact angle goniometer (KRUSS, Germany). First, cable samples were cut into 1 cm × 1 cm square specimens. Their surfaces were then wiped clean with anhydrous ethanol and a dust-free cloth to remove any oil and impurities. Following cleaning, the specimens were placed in a desiccator at room temperature for 24 h to ensure the surfaces were in a dry and stable state.

During the test, a 2 μL deionized water droplet was deposited onto the specimen surface using a microliter syringe. The instrument's high-speed camera automatically captured the shape of the droplet as it reached equilibrium on the solid surface, and specialized software (KRUSS DSA software) was used to calculate the static contact angle by quantifying the tangent angle of the droplet with the solid surface. To ensure data reliability, each specimen was measured at a minimum of three different locations, and the average value was taken as the contact angle for that sample.

### Halogen moisture analyzer measurement

To characterize the moisture content of the cable insulation layer, we selected samples from uncontaminated cables as well as those contaminated for 15, 30, 45 and 60 days. The test was conducted using a HD-100A halogen moisture analyzer. For each sample, we cut a 3–6 g piece of the insulation layer and placed it in the analyzer for moisture content determination. The test temperature was set to 105℃ in automatic mode, which automatically stops heating when the sample mass fluctuation is less than 0.5% to ensure a stable result. Finally, the moisture content of each sample was recorded for data analysis.

### FTIR analysis

To characterize the chemical structural changes of cable samples before and after contamination, we performed FTIR spectroscopy using a NEXUS 670 FTIR spectrometer (Thermo Fisher Scientific, USA). The experimental samples included an uncontaminated control group and an experimental group with a contamination period of six months.

Before analysis, the sample surfaces were cleaned with anhydrous ethanol and subsequently dried under vacuum at room temperature to completely remove surface moisture and residual liquid from the culture environment, ensuring that spectral signals in the hydroxyl region were not confounded by absorbed water. The measurements were conducted using Attenuated Total Reflectance (ATR) mode, which does not require additional sample preparation for solid materials. The spectral data were collected over a wavenumber range of 4000–400 cm^−1^ with a resolution of 4 cm^−1^ and 32 scans. A background spectrum was collected before each sample run for baseline correction to eliminate environmental interference.

After data acquisition, all spectra were normalized to the C-H stretching vibration peak at approximately 2920 cm^−1^ to allow for quantitative comparison of peak intensities. Peak fitting was performed using a Gaussian function to deconvolute overlapping bands and accurately determine peak positions and areas. By analyzing the FTIR spectra, we were able to identify changes in the functional groups on the sample surfaces and thus evaluate the effect of microbial contamination on the chemical structure of the cable material.

### XPS analysis

To analyze the changes in the elemental composition and chemical bonding states on the cable sample surfaces, we performed X-ray photoelectron spectroscopy (XPS) on an uncontaminated control group and an experimental group contaminated for six months. The analysis was conducted using an AXIS Supra XPS (Kratos, Shimadzu, Japan) under ultra-high vacuum (UHV) conditions. Before testing, all samples were cut into small pieces and mounted onto a sample stage. The X-ray source used for the analysis was Al Kα, with a source energy of 1486.6 eV.

First, a survey scan was performed to determine the elemental composition of the sample surface, with the pass energy set at 160 eV. Subsequently, high-resolution scans were collected for specific elements of interest (C, O, and Cl) at a pass energy of 20 eV to enable a detailed analysis of their chemical bonding states. All spectra were calibrated using the C 1s peak at 284.8 eV for charge correction. Through XPS analysis, we were able to quantitatively determine the elemental composition of the sample surfaces and reveal the impact of microbial contamination on the cable material at the level of chemical bonding.

### LCR digital bridge measurement

To characterize the changes in the electrical properties of the cable before and after contamination, a TH2816B LCR digital bridge was used. This experiment selected an un-contaminated healthy cable as the control group and a cable inoculated with *Curvularia lunata B3* and incubated for six months as the experimental group.

Before testing, a section of the outer sheath was carefully removed from one end of the cable with pliers to expose approximately 3 cm of the copper core. Subsequently, starting about 1 cm below the exposed copper core, copper tape was wrapped around the outer sheath to ensure the contact surface was fully covered. This also maintained an adequate distance between the copper tape and the exposed core to prevent surface leakage current.

After warming up the bridge for 30 minutes, the test voltage was set to 1 V. The positive terminal was connected to the exposed copper core, while the negative terminal was connected to the copper tape wrapped around the cable. In Cs-Rs test mode, the measurement frequencies were set to 1 kHz, 10 kHz, and 100 kHz, with the “SLOW” setting selected to ensure stable readings. Once the values on the display stabilized, the Cs (equivalent series capacitance) and Rs (equivalent series resistance) values were recorded for each frequency to evaluate the changes in the cable's electrical performance.

## Results and discussion

### Results

#### Microbial community composition and key species screening.

**Fig 1:**
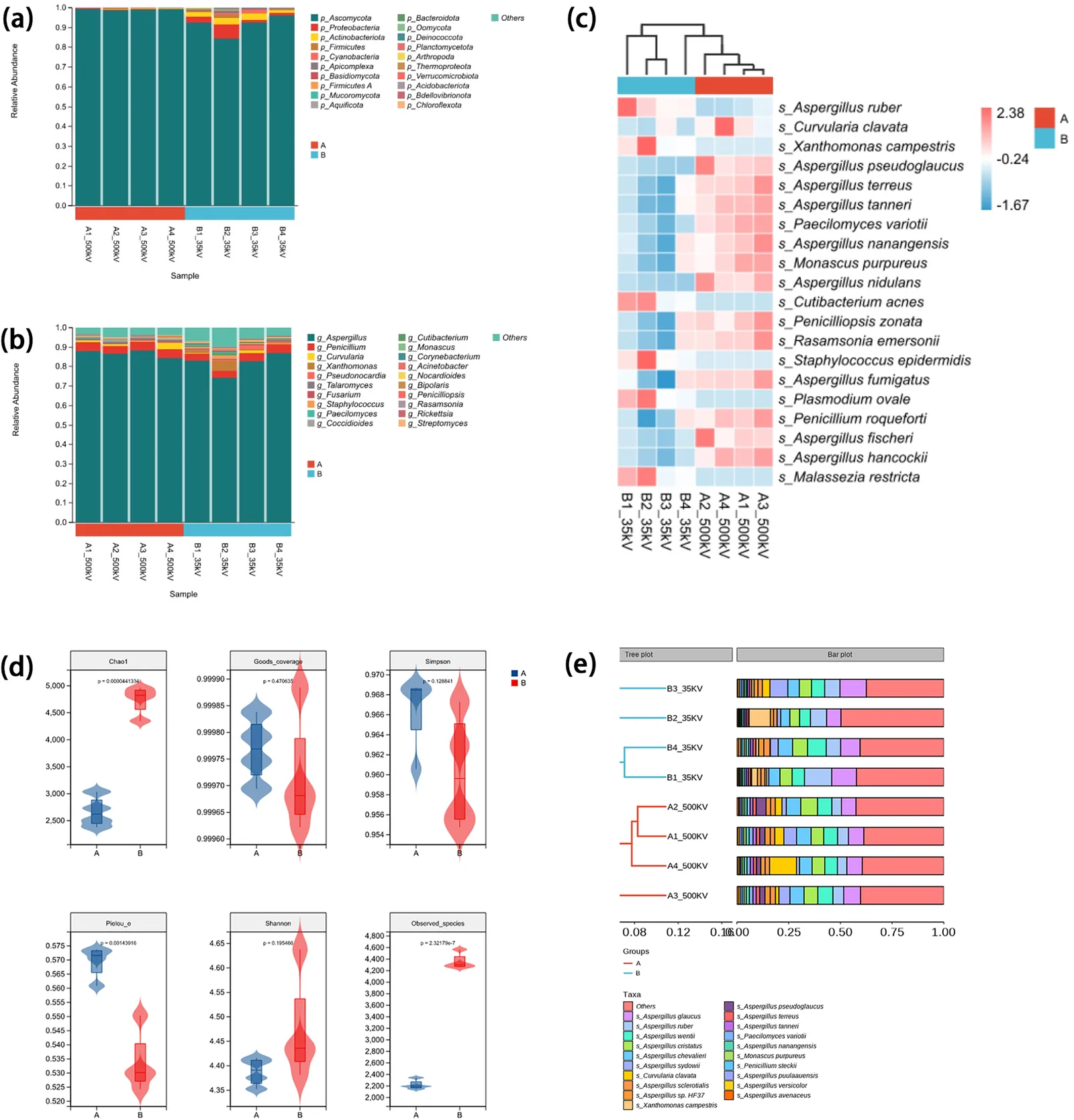
Analysis of the composition of microbial communities and differences in key species in contaminated cables of substation. (a): Composition analysis diagram of microbial communities in contaminated cable samples from substations at the phylum level; (b): Composition analysis of microbial communities in contaminated cable samples from substations at the genus level; (c): Heat map for differential analysis of key microbial species in contaminated cable samples from substations; (d): Hierarchical clustering analysis of microbial community beta diversity in contaminated cable samples from substations across different voltage classes; (e): Species-level composition and metagenomic annotation of key microbial taxa (Curvularia) in contaminated cable samples from substations.

Fig 1a illustrates the ecological dominance of Ascomycota at the phylum level across all samples, with its relative abundance exceeding 90% universally. Following Ascomycota, Proteobacteria and Actinobacteria emerge as pivotal constituents of the microbial community in substation cable pollution. Notably, while other phyla such as Firmicutes and Cyanobacteria are present, their relative abundance remains minimal. These findings underscore a relatively uncomplicated microbial community structure on the surface of substation cables, primarily governed by a select few dominant fungi and bacteria.

In Fig 1b, the broad-level composition of microbial communities on cable surfaces is depicted for different voltage classes (Group A representing the 500kV sample group and Group B denoting the 35 kV sample group). The outcomes indicate the prevalence of *Aspergillus* across all samples, comprising over 70% of the total composition. *Aspergillus* emerged as the predominant member of the microbial community in the studied environment, with other genera such as *Penicillium*, *Curvularia*, *Xanthomonas*, and *Pseudocardia* also exhibiting notable abundance levels. These genera collectively represent the primary microbial groups in the ecosystem affected by substation cable pollution.

To further evaluate the richness, diversity, and evenness of these microbial communities, alpha diversity analysis was performed (Fig 1c). The results demonstrate that the sequencing depth was sufficient to fully cover the vast majority of species present in the samples, as evidenced by the Good's coverage index approaching 100% across all samples, ensuring the reliability of subsequent analyses. Regarding species richness, the Chao1, ACE, and Observed species indices collectively reveal that Group B (35 kV) possesses a significantly higher number of species compared to Group A (500 kV). This supports the premise that differences in voltage class exert an influence on the overall species richness of the microbial communities. In terms of species diversity, however, the Simpson and Shannon indices show no significant differences between the two groups. This suggests that while Group B exhibits higher richness, the additional species are likely low-abundance, rare taxa that have a limited impact on the overall community structure. This observation aligns with the species evenness reflected by the Pielou's evenness (e) index, which indicates similar evenness levels between the two groups, both being heavily dominated by a few highly abundant taxa. Taken together, while the different voltage-class environments alter the species richness of the microbial communities, they do not fundamentally shift the overall diversity and evenness. This indicates a structural characteristic where Group B is “richer but less even,” whereas Group A is “less rich but more even,” potentially linked to the ecological dynamics of a few highly adaptive key species dominating this specific environment.

Hierarchical clustering analysis was subsequently employed to resolve the structural similarities among the microbial communities from different voltage classes (Fig 1d). The clustering based on Bray-Curtis distance shows that all sampling sites are initially split into two distinct, independent branches corresponding to the 500 kV group (Group A) and the 35 kV group (Group B). This clear separation indicates that the voltage class is a key environmental driver shaping the microbial community structure on control cable surfaces. Within the 500 kV group, the short clustering distances between samples reflect high compositional consistency. This suggests that the high-voltage electric field exerts a strong, directional selective pressure on the microbial communities, driving them to evolve toward a highly specialized state. Combined with the species abundance profile, the dominant fungi occupy a prominent and stable proportion in Group A, whereas the community composition in Group B exhibits higher heterogeneity and diversification. This beta diversity-based clustering differentiation strongly demonstrates the ecological selection effect of electric field intensity on microbial colonization. To pinpoint the specific microbial groups driving these performance changes and identify candidates with strong environmental tolerance within this selective environment, a differential abundance analysis was conducted.

The results, depicted in Fig 1c, visually illustrate the relative abundance shifts of dominant species. Samples distinctly clustered into two groups corresponding to the 500 kV and 35 kV substation environments. Notably, *Curvularia* showed significant enrichment in the 500 kV samples, as evidenced by darker and more intense red color blocks compared to the lighter blocks observed in the 35 kV samples. Although metagenomic profiling annotated this taxon as *Curvularia clavata* (as shown in Fig 1e), subsequent culture-dependent isolation from the field samples yields dominant fungal strains identified as *Curvularia lunata* through morphological observation and ITS rRNA gene phylogenetic analysis. This discrepancy is common in environmental metagenomics due to the high sequence similarity among closely related *Curvularia* species, which limits the taxonomic resolution of short-read metagenomic sequencing at the species level, alongside potential reference database annotation biases. Consequently, we rely on the robust genus-level classification of the metagenomic data to identify *Curvularia* as the key group associated with high-voltage cable pollution. These consistent findings from both metagenomic and culture-dependent approaches firmly support the selection of *Curvularia lunata* as a representative model organism for investigating the impact of microbial contamination on cable material performance.

### Morphological characteristics of *Curvularia lunata*

**Fig 2:**
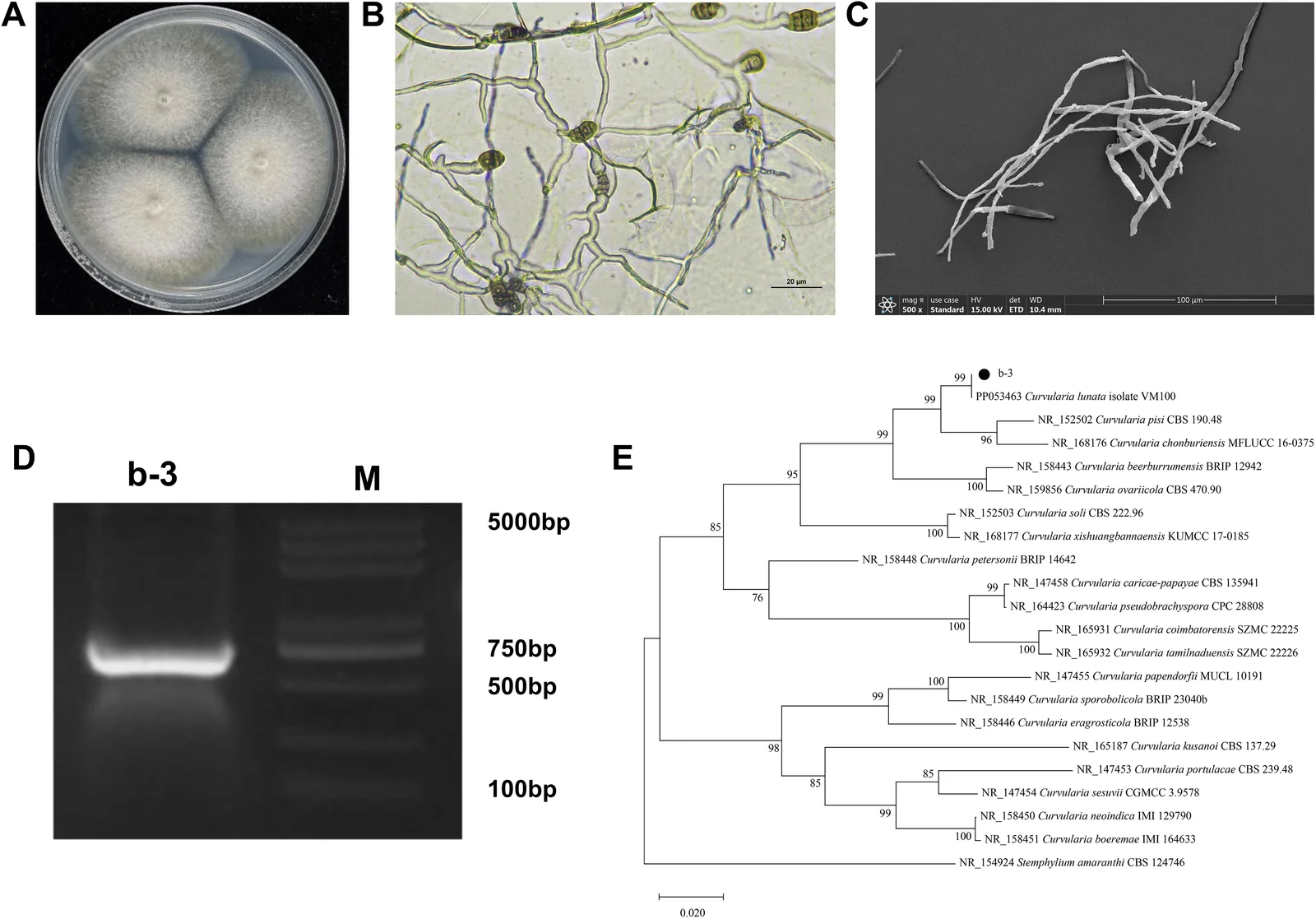
Morphological characteristics of *Curvularia lunata.* (a): Colony morphology on PDA medium; (b): Microscopic morphology showing conidia and hyphae; (c): Scanning electron microscopy (SEM) image of hyphal morphology; (d): Agarose gel electrophoresis of PCR-amplified ITS region from strain *B3*; (e): Neighbor-joining phylogenetic tree based on ITS rDNA sequences showing the taxonomic position of strain *B3*.

Fig 2a displays the colony characteristics of *Curvularia lunata* cultivated on PDA medium. The colonies exhibit a villous or flocculent appearance, with gray hyphae that transition to black as growth progresses. In Fig 2b and 2c, the microscopic features of *Curvularia lunata* are depicted under microscope. The elongated, branched, septate mycelium is prominently visible. The conidia are dark brown, possess distinct septa, typically consist of three to four cells, with the central cell being slightly larger than the terminal cells, displaying a distinctive curved shape resembling a crescent. This distinctive morphology is a hallmark of *Curvularia lunata*.

At the molecular level, genomic DNA of strain *B3* was amplified by PCR using the universal fungal primers ITS1/ITS4 (Fig 2d), yielding a target band of approximately 600 bp. The resulting sequence was submitted to the NCBI database for BLAST alignment. Subsequently, a phylogenetic tree was constructed using the Neighbor-joining method in MEGA 11.0 (Fig 2e). Phylogenetic analysis revealed that strain *B3* clustered with *Curvularia lunata* isolate VM100 (Accession No.: PP053463) with high bootstrap support. Collectively, based on both the morphological characteristics and the molecular phylogenetic analysis, strain *B3* was definitively identified as *Curvularia lunata*.

### SEM analysis: Cable surface morphology

To investigate the impact of microbial erosion on cable material microstructure and its progression, we systematically examined the surface morphology of short cable insulation contaminated with *Curvularia lunata* over varying durations using scanning electron microscopy (SEM).

**Fig 3:**
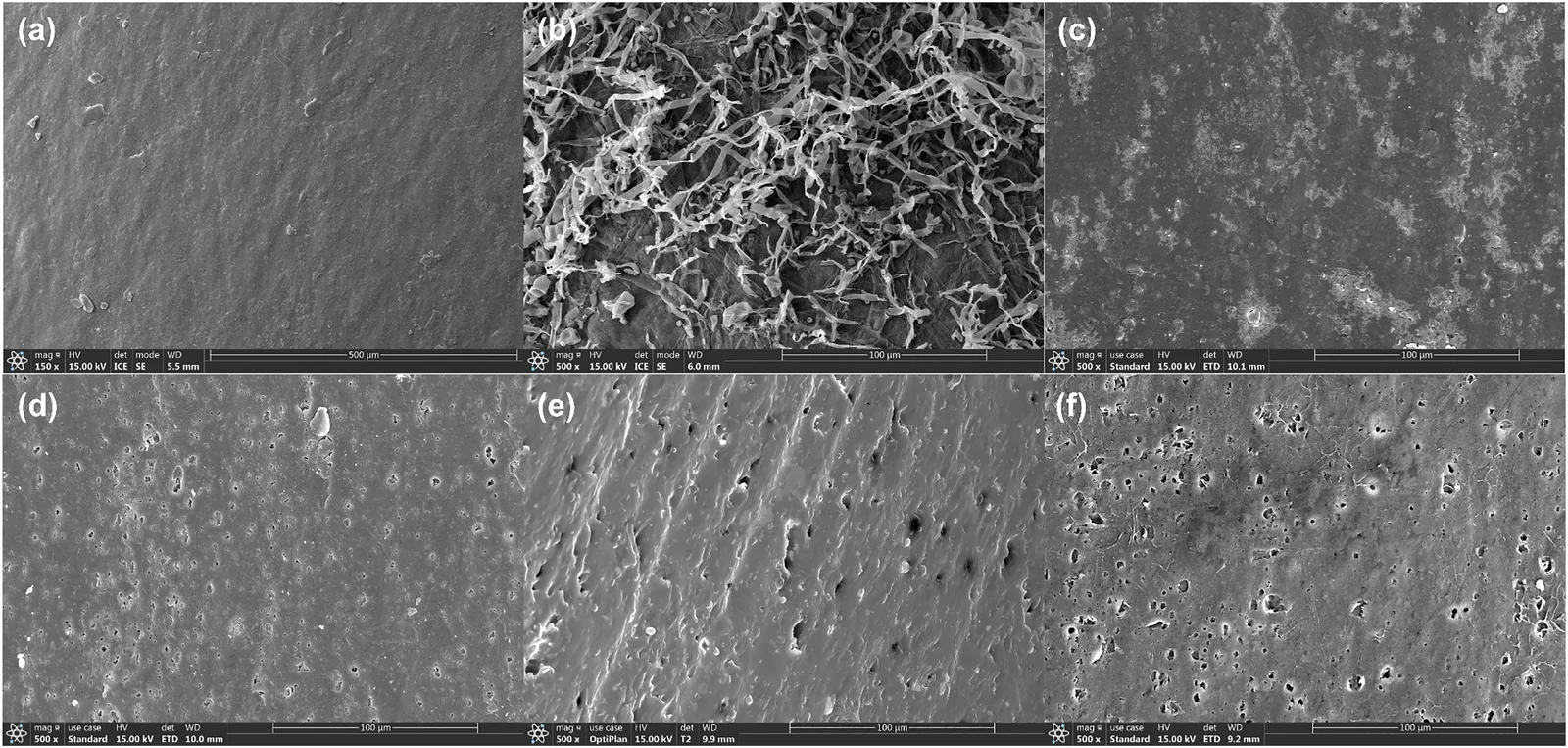
SEM images of the surface of short cable samples. (a): Surface morphology of the control group cable; (b): Adhesion state of the mycelial network on the cable surface; (c): Surface morphology of the cable contaminated by *Curvularia lunata* for 3 months; (d): Surface morphology of the cable contaminated by *Curvularia lunata* for 6 months; (e): Surface morphology of the cable contaminated by *Curvularia lunata* for 9 months; (f): Surface morphology of the cable contaminated by *Curvularia lunata* for 12 months.

Fig 3a illustrates the pristine surface of cable insulation in the control group, devoid of microbial contamination, displaying a notably flat and smooth profile without evident physical defects like cracks or biological adhesions. The surface roughness (Ra) is anticipated to be at a submicron scale, indicative of material structural integrity.

In Fig 3b, the initial colonization and formation of hyphal networks by *Curvularia lunata* filaments on the cable insulation surface are clearly depicted. The slender, filamentous mycelium typically ranges from 1–3 µm in diameter, intricately interwoven and densely adhered to the material surface, establishing an early biofilm structure. This observation signifies the successful attachment of *Curvularia lunata* and the commencement of biointeractions with the cable insulation.

Fig 3c illustrates the surface morphology of cable insulation following three months of exposure to *Curvularia lunata* contamination. The images depict localized color fading, distinct roughening of the material's surface, irregular mottled areas, and subtle depressions ranging from 0.5 to 2 µm in size. These initial alterations likely stem from the degradation of the insulation surface's molecular structure, potentially induced by extracellular polymers (EPS) or microbial metabolites, leading to diminished surface gloss and uniformity.

Subsequent degradation of the cable insulation surface is evident in Fig 3d, six months post-contamination. Scanning electron microscopy (SEM) images reveal an increased presence of fine holes and pits of varying sizes (typically 2–5 µm in diameter) on the material's surface. The emergence of these microscopic defects suggests a progression in *Curvularia lunata* erosion from mere surface adhesion to localized penetration and material matrix destruction, possibly attributed to microbial enzyme decomposition or the corrosive action of acidic metabolites.

Fig 3e illustrates substantial surface damage to the cable insulation nine months post-contamination. The observed holes in the image notably enlarge, reaching diameters of 5–15 µm, while interconnected gullies typically measure 3–10 µm in width. Moreover, evident spalling and microcracks are visible in certain areas of the insulation material, with microcrack lengths extending to tens of microns. These profound structural alterations suggest that microbial erosion has inflicted significant harm on the internal composition of the insulation layer, severely compromising the material's integrity and continuity.

In Fig 3f, after a contamination period of up to twelve months, the cable insulation surface exhibits extremely severe macroscopic and microscopic damage. The holes continue to expand, coalescing to form erosion pits and intricate deep trenches measuring 10–30 µm or larger. The material's surface structure becomes notably rough and loose, accompanied by extensive cracking and peeling, with the peeling layer thickness potentially reaching several microns. These features strongly indicate that prolonged microbial erosion has caused irreversible structural deterioration and substantial performance decline in cable insulation materials, posing a risk of insulation failure.

### WCA analysis: Cable surface wettability

**Fig 4:**
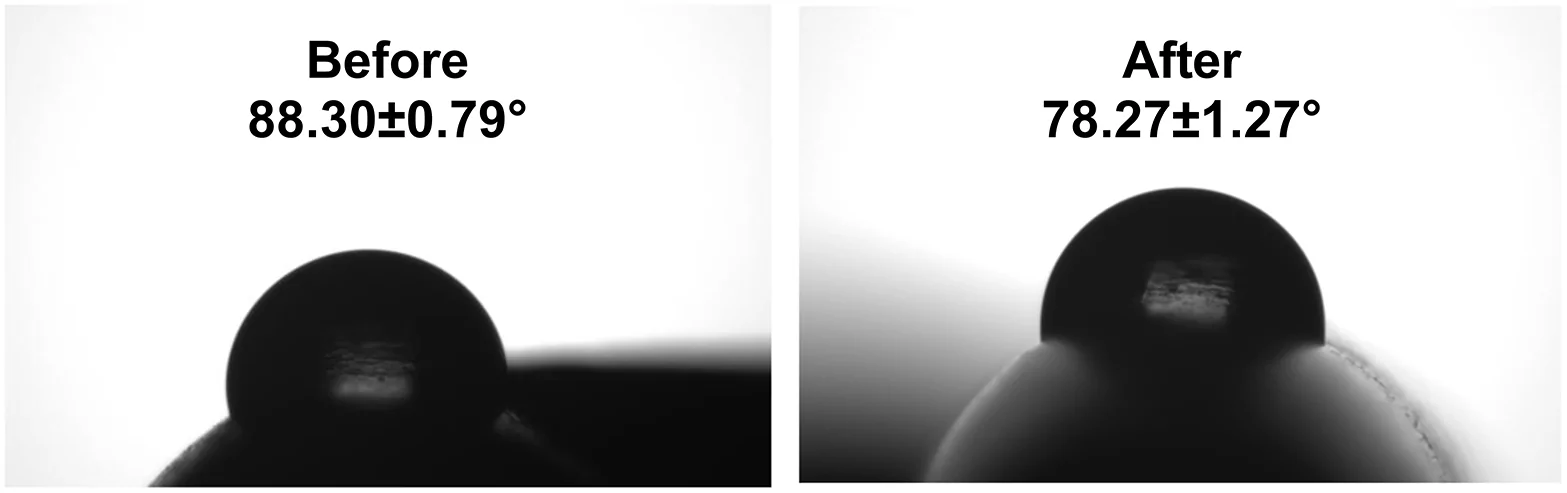
Diagram of changes in water contact angle on the surface of cable insulation layer before and after contamination by *Curvularia lunata.* (a):Water contact angle on the surface of cable insulation layer in the control group; (b):Water contact angle on the surface of cable insulation layer in the contaminated group.

The Water Contact Angle Test (WCA) is a common method for assessing alterations in surface hydrophobicity. As shown in Fig 4, initially, the cable insulation layer exhibited robust hydrophobicity in the control group, as evidenced by water droplets forming complete spheres on the surface, resulting in a water contact angle of 88.30 ± 0.79°(n = 6). Following six months of exposure to *Curvularia lunata* contamination, the water contact angle decreased significantly to 78.27 ± 1.27° (n = 6). A two-sample t-test was performed to compare the mean contact angles between the two groups. The results showed a statistically highly significant difference (t = 12.13, *P* < 0.001), with a very large effect size (Cohen's d = 7.00), indicating a notable decline in hydrophobicity. This decrease led to partial spreading of water droplets on the surface, suggesting that *Curvularia lunata* had eroded the cable insulation layer to some extent. This erosion may result in an increase in polar groups on the material's surface or alterations in surface roughness, consequently enhancing surface energy and hydrophilicity.

### Moisture analysis: Cable moisture characteristics

**Fig 5:**
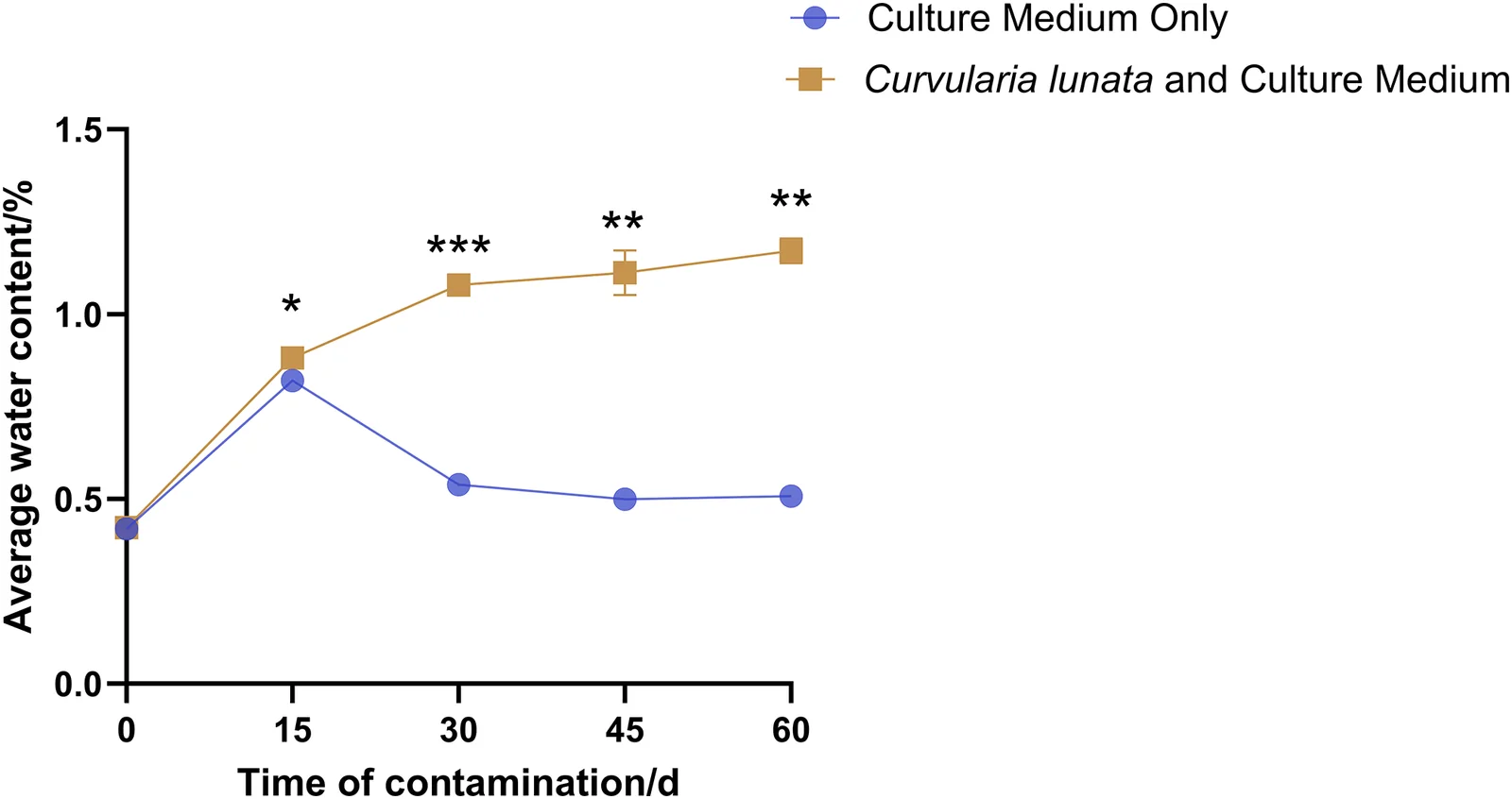
The average water content of cable samples over time with and without *Curvularia lunata* inoculation. Data are presented as mean ± SD (n = 3). Error bars for some data points are smaller than the symbol size.

Moisture content analysis is commonly employed to assess the moisture absorption characteristics of materials, the extent of moisture exposure, and the influence of microorganisms on material microenvironments. In this investigation, cable samples’ moisture content was periodically assessed using a halogen moisture meter to investigate the impact of *Curvularia lunata* on cable moisture properties. The results are shown in Fig 5. Initially, the moisture content of short cable samples in the control group, which were solely exposed to the medium, exhibited a rapid increase during the initial phase of the experiment (0–15 days), reaching a maximum value of approximately 0.8% on the 15th day. This surge can be attributed to the direct influx of external moisture from the medium. Subsequently, moisture gradually evaporated over time, leading to a swift decline in the water content of these samples post-15 days, stabilizing at around 0.5% after 30 days. Following the inoculation of *Curvularia lunata* and the addition of culture medium, the experimental group's moisture content also experienced a rapid rise from to 15 days. However, unlike the control group, the moisture content did not decrease after 15 days but continued to exhibit a gradual upward trajectory. The peak moisture content for this set of samples was observed at approximately 1.2% after 60 days.

The comparative findings between the two groups unequivocally demonstrate the significant influence of *Curvularia lunata* on the alteration of cable water content. This fungus not only adheres to the cable's surface but also facilitates water retention and suppresses evaporation through its mycelial growth. Furthermore, its metabolic processes generate water, thereby maintaining the cable in a prolonged state of elevated humidity. This persistent moisture elevation induced by fungal actions could potentially jeopardize the cable's insulation efficacy and operational longevity.

### FTIR & XPS analysis: Cable chemical structure

**Fig 6:**
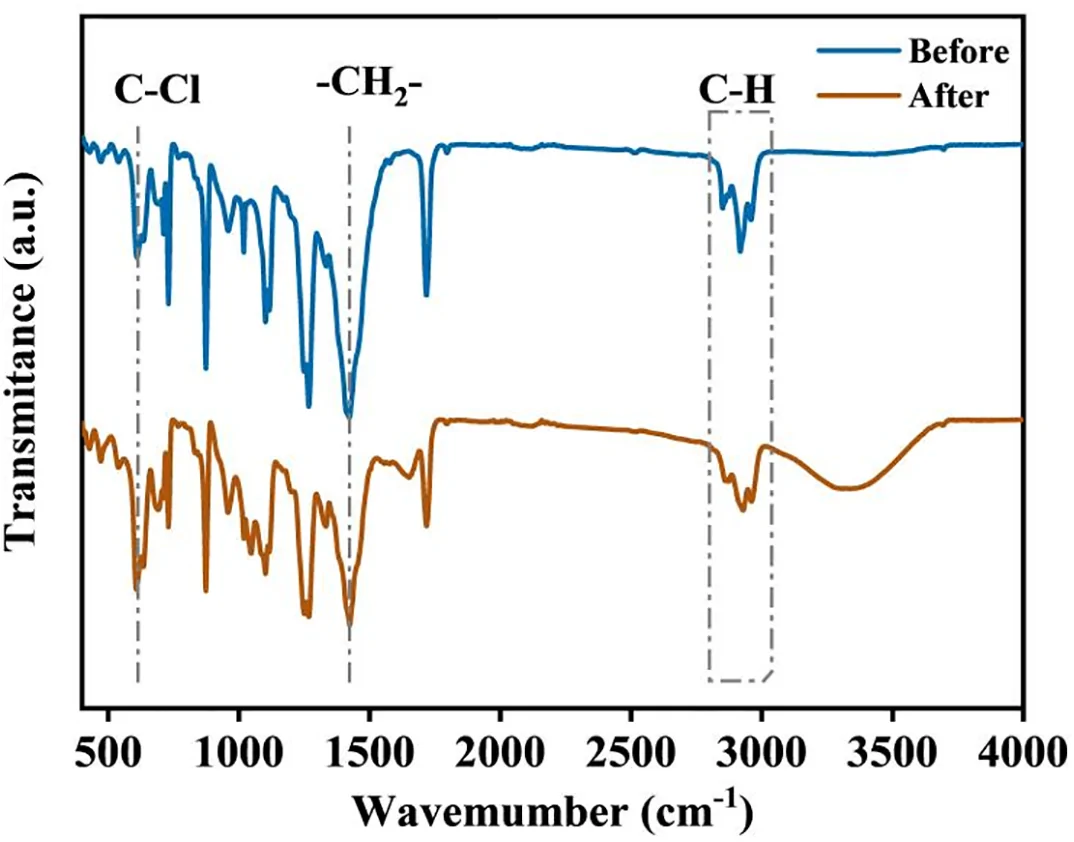
FTIR spectra of cable insulation material before and after microbial contamination (normalized to the C-H peak at 2920 cm^-1^).

To assess the impact of *Curvularia lunata* on cable insulation materials, FTIR was employed to examine the surface of cables pre- and post-contamination. The cable's outer layer primarily comprises polyvinyl chloride (PVC). Analysis, depicted in Fig 6, illustrates marked distinctions in the spectra of uncontaminated and contaminated cables, elucidating alterations in material chemistry induced by this strain. Initially uncontaminated samples exhibit characteristic PVC absorption peaks: signals at approximately 2920 cm^−1^ and 2850 cm^−1^ correspond to C-H stretching vibrations of the -CH2- moiety within the polymer chain, while a prominent peak at around 620 cm^−1^ signifies the presence of the C-Cl bond, indicative of the material's original chemical composition.

Following contamination with *Curvularia lunata*, the cable spectrum underwent significant alterations. Notably, the absorption peak of the C-Cl bond at approximately 620 cm^−1^ experienced substantial attenuation or complete disappearance. This phenomenon signifies that the biological actions of this strain induce the dechlorination of PVC polymer chains and the breakdown of C-Cl bonds. Furthermore, the metabolic processes of this strain may induce modifications in other chemical structures, such as the emergence of a novel broad absorption peak around 3400 cm^−1^ in the spectrum. This peak could be ascribed to the generation of O-H groups or the adsorption of water molecules, indicating the development of biofilms or the introduction of fresh oxygen-containing functional groups.

**Fig 7:**
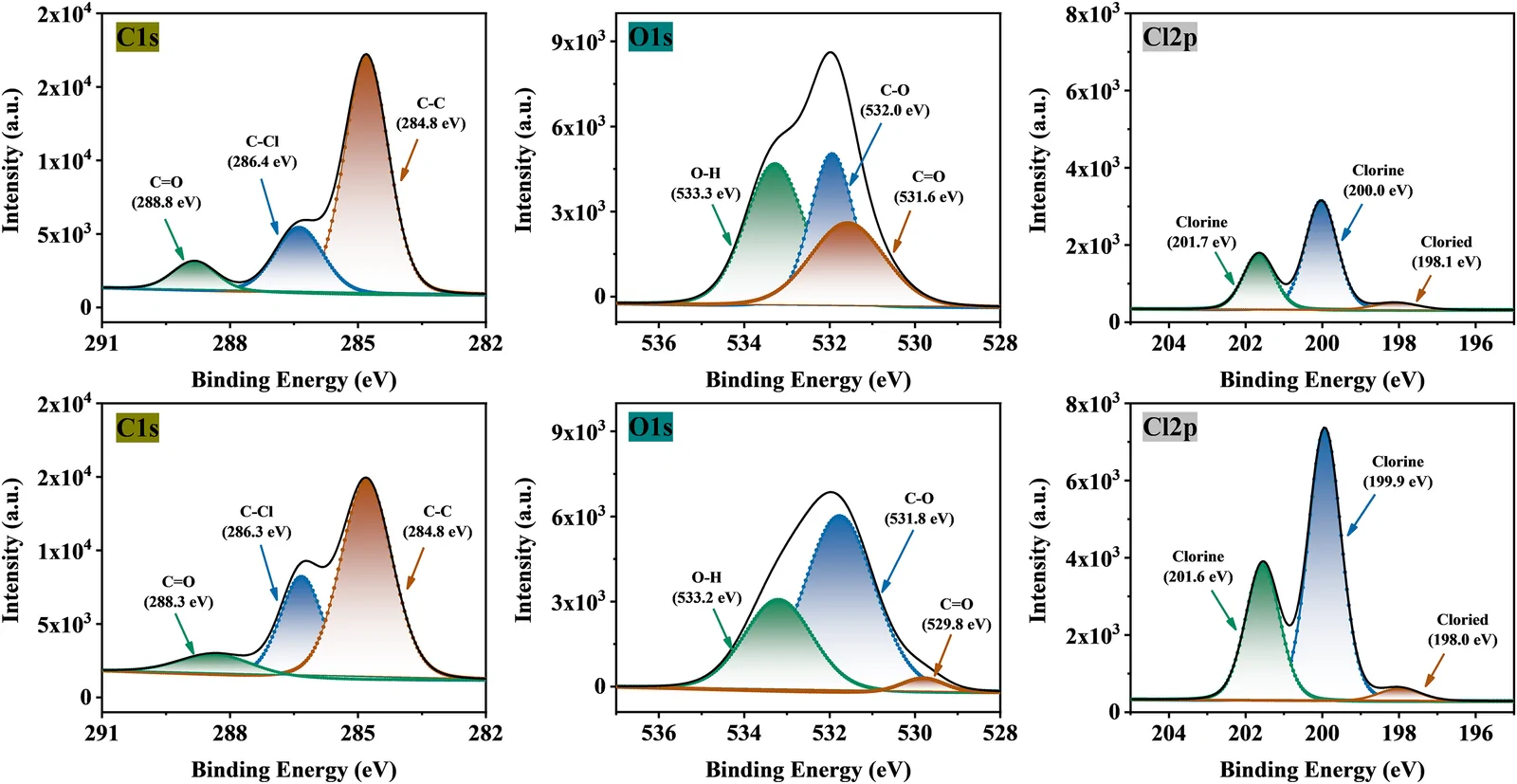
The XPS spectra of the cable insulation layer surface before and after contamination by *Curvularia lunata.*

To thoroughly investigate the impact of *Curvularia lunata* contamination on the chemical composition of cable insulation material surfaces, X-ray photoelectron spectroscopy (XPS) was employed to analyze the elemental valence states and chemical bonds present on the surfaces of pristine cables in the control group and those subjected to six months of *Curvularia lunata* contamination in the experimental group. The outcomes are depicted in Fig 7, which displays high-resolution XPS spectra of C1s, O1s, and Cl2p on the cable surfaces of the control group (upper row) and the experimental group (lower row), elucidating the chemical valence distribution of each element via peak fitting.

The C1s spectrum of the control cable exhibits two prominent peaks at approximately 284.8 eV for C-C/C-H bonds and 286.3 eV for C-Cl bonds, consistent with the characteristic carbon structure of polyvinyl chloride (PVC). The O1s spectrum indicates minimal oxygen content or slight adsorption of oxygen, while the Cl2p spectrum displays typical bimodal features of C-Cl bonds (Cl2p3/2 at around 199.7 eV and Cl2p1/2 at about 201.3 eV). Conversely, contamination of the cables by *Curvularia lunata* induces notable changes in the XPS spectra: a new peak emerges at approximately 288. eV in the C1s spectrum, typically associated with carbonyl carbon (C = O) or carbon-oxygen double bonds (C-O). Simultaneously, the intensity of the O1s spectrum markedly increases, indicating the presence of hydroxyl groups (-OH, indicated by a green arrow) and carbonyl oxygen (C = O, indicated by a blue arrow). Moreover, the intensity of the C-Cl bond peak in the Cl2p spectrum decreases (indicated by an orange arrow), potentially accompanied by the appearance of a new peak attributed to inorganic chloride ions (Cl-, indicated by a green arrow).The observed alterations suggest that surface oxidation of the cable due to crescent mold contamination generates oxygen-containing functional groups, indicating potential dechlorination of the PVC material wherein chlorine atoms detach from the polymer chain, potentially manifesting as chloride ions.

X-ray photoelectron spectroscopy (XPS) analysis findings offer compelling evidence that the presence of *Curvularia lunata* can significantly modify the surface chemical composition of polyvinyl chloride (PVC) cable insulation. Specifically, the results indicate: 1) a notable increase in surface oxidation, characterized by the formation of oxygen-containing functional groups like carbonyl and hydroxyl groups; and 2) evident dechlorination reactions leading to a reduction in surface C-Cl bonds. These alterations in chemical structure serve as direct confirmation of the biodegradation and chemical deterioration of cable insulation materials by *Curvularia lunata*. This observation aligns well with the morphological variations observed through scanning electron microscopy (SEM) and the decrease in hydrophobicity as demonstrated by water contact angle measurements. Collectively, these findings elucidate the microscopic mechanism by which microbial contamination contributes to the degradation of cable performance.

### LCR bridge analysis: Cable electrical performance

**Fig 8:**
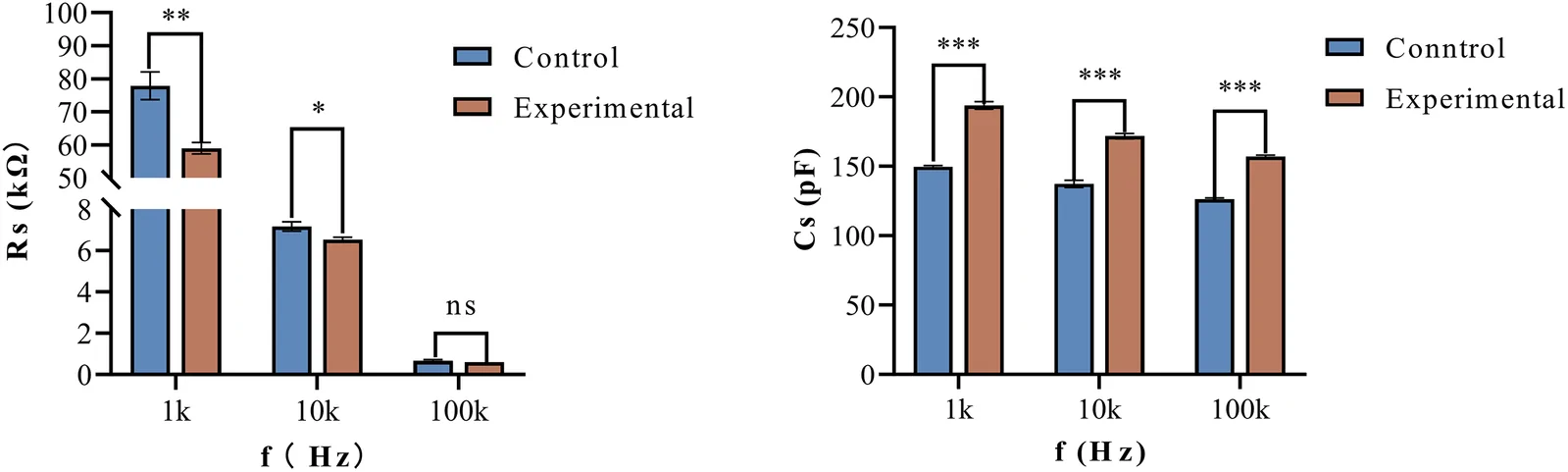
The impact of *Curvularia lunata* contamination on the electrical properties of cable insulation layers. (a):Variations in the series resistance (Rs) of cables with frequency before and after *Curvularia lunata* contamination; (b):Variations in the series capacitance (Cs) of cables with frequency before and after *Curvularia lunata* contamination.

This study assessed the electrical performance of cables contaminated with *Curvularia lunata* over a six-month period compared to uncontaminated control cables. The evaluation was conducted using an LCR digital bridge, with results presented in Figs 8a and 8b. Measurements of series resistance (Rs) and series capacitance (Cs) at frequencies of 1 kHz, 10 kHz, and 100 kHz revealed notable disparities in electrical behavior between the contaminated cables (experimental group) and the healthy cables (control group), suggesting that the long-term colonization of *Curvularia lunata B3* can alter the AC impedance and dielectric response of the PVC cable insulation layer.

As illustrated in Fig 8a, the Rs values of the experimental group were lower than those of the control group across all tested frequencies. This difference was highly pronounced at 1 kHz (*P < 0.01*), remained statistically significant at 10 kHz *(P < 0.05*), and became non-significant at 100 kHz (*P > 0.05*). This frequency-dependent behavior indicates that the fungal contamination predominantly impacts the low-frequency electrical response. The formation of a fungal biofilm, coupled with the secretion of organic acids, extracellular enzymes, and other polar metabolites, likely promotes moisture absorption and alters the chemical composition and microstructure of the PVC surface. The accumulation of moisture and mobile ions enhances ion migration and interfacial polarization under low-frequency electric fields, leading to reduced Rs. At higher frequencies, interfacial charges and mobile ions fail to track the rapidly alternating field, thereby diminishing the disparity between the two groups at 100 kHz.

Capacitance analysis (Fig 8b) revealed that the capacitance (Cs) values of the contaminated cables were significantly higher than those of the control group across all tested frequencies (*P < 0.001*), demonstrating a highly consistent impact on dielectric properties. The elevated Cs suggests an increase in the effective dielectric constant or the degree of interfacial polarization within the insulation system. The accumulation of polar metabolites, biofilm, and adsorbed water on the PVC surface and within micro-defects enhances the material's polarizability. Furthermore, the heterogeneous interfaces formed among the fungal hyphae, biofilm, water, and PVC induce more pronounced interfacial polarization, thereby increasing the measured equivalent capacitance. These findings corroborate material characterization results, including reduced contact angles, increased moisture content, surface pitting, and the proliferation of oxygen-containing functional groups.

Since increased capacitance lowers capacitive reactance, the contaminated cables exhibit lower AC impedance and a stronger current response under identical frequency and AC voltage conditions. Consequently, the concurrent decrease in Rs and increase in Cs indicate that the long-term colonization of *Curvularia lunata B3* alters the AC conductive and dielectric behaviors of the PVC insulation, compromising its signal-blocking efficacy.

In summary, LCR digital bridge testing demonstrates that *Curvularia lunata B3* contamination significantly reduces the series resistance and increases the series capacitance of PVC cables, leading to diminished AC impedance and enhanced dielectric response. These shifts indicate that the electrical isolation performance of the insulation layer has been adversely affected. In the long term, such drift in electrical parameters poses a potential threat to the operational stability of substation control cables by elevating the risks of signal anomalies and further insulation degradation. Notably, as Rs represents an AC equivalent parameter rather than DC insulation resistance, these results confirm the degradation of AC electrical performance; however, further investigation via dedicated insulation resistance and leakage current tests is required to verify reductions in DC resistance and increases in leakage current.

## Discussion

Over prolonged operation, particularly in challenging substation environments characterized by elevated temperatures and humidity, secondary cable insulation is vulnerable to microbial corrosion, a significant factor impacting their longevity and dependability [5]. This study integrated metagenomic analysis with multidimensional characterization to elucidate the erosion mechanisms of *Curvularia lunata* on cable insulation.

Metagenomic sequencing identified *Curvularia lunata* as a dominant species in high-voltage (500 kV) environments. This discovery establishes a biological rationale for designating *Curvularia lunata* as a model organism, hinting at its adaptation to high-stress environments or its pivotal role in material deterioration [10]. The erosion process, visualized via SEM, progressed from initial hyphal adhesion to deep pitting and spalling over 12 months. These microscopic changes vividly illustrate the complete microbial erosion process, spanning from surface colonization to profound material matrix degradation [11].

This physical damage was intrinsically linked to chemical and physical property changes. The decline in hydrophobicity, evidenced by the significant decrease in WCA, signifies an increase in surface energy and polarity, likely facilitating the adsorption and infiltration of water molecules [12]. Concurrently, moisture analysis confirmed that the hyphal network facilitates water retention and suppresses evaporation, maintaining the cable in a prolonged state of high humidity [13].

FTIR and XPS analyses further revealed the chemical mechanism: *C. lunata* enzymatic activity drives the dechlorination of PVC chains and introduces oxygen-containing functional groups (C = O, -OH) [13,14]. This chemical breakdown, combined with moisture accumulation and physical defects, culminated in a significant decline in electrical performance. The LCR bridge results showed decreased series resistance (Rs) and increased capacitance (Cs), indicating compromised electrical isolation performance and an elevated risk of electrical malfunction [15].

To decipher the potential biochemical mechanisms associated with the observed surface alteration of PVC, our experimental findings should be interpreted cautiously alongside established polymer biodeterioration literature. Although the specific enzyme profile of *C. lunata B3* was not directly characterized here, a distinct *C. lunata* isolate (MY3) has been reported to produce an extracellular laccase capable of catalyzing oxidative single-electron transfers [16]. In fungal–polymer interactions, extracellular oxidative enzymes may contribute to the oxidative modification of susceptible polymer surfaces; however, their specific contribution to PVC dechlorination remains to be verified [16]. This cautious interpretation aligns with our FTIR and XPS spectral data—showing an increase in oxygen-containing functionalities and an attenuation of C–Cl-related surface signals—as well as previous studies reporting structural alterations in the HCCl region and molar mass changes of PVC upon fungal exposure [17]. Concurrently, non-targeted metabolomic profiling detected enriched phthalate-related features at the cable surface. Given that phthalate plasticizers (e.g., DEHP) are non-covalently incorporated into flexible PVC matrices [18], these metabolomic signals are compatible with possible migration, redistribution, and/or transformation of plasticizer-associated components within the bio-interface [18]. Furthermore, in other fungal–polymer systems, organic-acid secretion has been associated with the interfacial deterioration of hydrolysis-susceptible polymer components, such as polyurethane coatings containing ester and urethane bonds [19]. Taken together, these physical defects (e.g., microscopic holes and furrows observed via SEM) and surface chemical modifications reflect a multifaceted deterioration process, providing a plausible framework for the observed loss of electrical insulation performance.

Placing these findings in the broader context of polymer biodeterioration offers relevant insights into the environmental persistence and material-interaction capabilities of *C. lunata*. Many studies of fungal polymer biodeterioration have used environmentally derived isolates under controlled laboratory cultivation conditions [20–22]. In contrast, our metagenomic survey identified *C. lunata* as a dominant fungal taxon on cables collected from a 500 kV substation, and the isolated strain B3 was able to colonize cable insulation under the experimental conditions. Melanin may represent one trait potentially relevant to environmental persistence. In *C. lunata*, DHN-melanin biosynthesis has been implicated in pigmentation and infection-related development, and melanin may contribute to fungal persistence under adverse conditions [23]. However, whether melanin-associated pathways are activated in strain B3 during cable colonization remains to be determined. Furthermore, in vitro studies have shown that a rotating magnetic field can alter the activity and kinetic properties of purified laccase from Trametes versicolor, with the magnitude and direction of the effect depending on field parameters [24]. Whether electromagnetic exposure at substations similarly affects fungal growth, enzyme secretion, or enzyme activity in *C. lunata B3* is unknown. Likewise, in the model filamentous fungus Magnaporthe oryzae, phospholipase C-mediated Ca² ⁺ flux is required for surface-responsive, infection-related morphogenesis, including appressorium formation [25]. Similar signaling modules may be relevant to fungal surface-associated development; however, their role in *C. lunata B3* colonization of cable insulation has not been examined. In a mixed-fungal culture–polyimide coating system, fungal colonization was associated with water and ion ingress, a progressive decline in coating resistance, and electrochemical evidence of polymer-integrity loss [26]. Although the fungal community and polymer type differ from those of the present system, these findings provide a relevant precedent for interpreting the electrical changes observed in PVC-based cable insulation. Collectively, these studies suggest several testable hypotheses regarding the persistence and surface-associated growth of *C. lunata B3* on cable insulation. Direct validation will require targeted analyses of melanin production, stress-response pathways, enzyme activity, and the effects of field-relevant electromagnetic exposure.

The concurrent decrease in series resistance (Rs) and increase in series capacitance (Cs) indicate that exposure to *C. lunata B3* modified both the PVC insulation matrix and the cable–environment interface. This response should not be interpreted as evidence of PVC-chain scission alone. In the present study, fungal colonization was accompanied by surface damage, while the appearance of polar -OH and C = O functionalities, the decrease in water contact angle to 78.27°, and the increase in moisture content to approximately 1.2% together indicate the formation of a more hydrophilic and water-accessible surface. Such changes are consistent with previous evidence that fungal exposure can induce surface deterioration and chemical structural changes in PVC [17]. A similar decrease in the electrical resistance of plasticized PVC cable insulation has also been reported following fungal colonization, where fungal metabolites were proposed to increase polymer conductivity [27]. Biofilm formation may provide an additional interfacial contribution. Microbial biofilms are common on humid material surfaces and consist of microbial cells embedded in extracellular polymeric material [28]. However, the present metabolomic data alone do not establish the composition, spatial localization, or electrical conductivity of an EPS layer formed by *C. lunata B3*. Therefore, a more conservative interpretation is that retained water, dissolved ions, and fungal metabolites at the colonized interface, together with moisture and ion ingress into the modified PVC surface, may enhance low-frequency ionic conduction and interfacial polarization. This interpretation is consistent with EIS observations in a mixed-fungal/polyimide coating system, in which an initial decline in coating resistance was associated with the ingress of water and ionic species into the polymeric matrix, followed by further fungal deterioration [26]. Accordingly, the observed Rs-Cs response is best considered an equivalent-circuit signature of coupled interfacial and matrix changes, rather than a direct measure of any single degradation process. Direct characterization of EPS, surface ions, and electrical properties before and after removal of the fungal biomass will be required to distinguish the contribution of the fungal interfacial layer from intrinsic PVC matrix degradation.

It is worth noting that while macroscopic measurements such as WCA (n = 6) and moisture content (n = 3) involved statistical replications, microscopic and spectroscopic analyses (SEM, FTIR, and XPS) were evaluated on representative specimens across multiple distinct sampling regions to ensure spatial uniformity and data reliability. Although these surface characterizations effectively capture the physical and chemical trends of fungal erosion, future studies incorporating multi-batch experimental replicates will further strengthen the quantitative robustness of these mechanistic insights.

We acknowledge that the present laboratory simulation utilized a rich nutrient medium (PDA) to cultivate *Curvularia lunata*, a setup that differs from the oligotrophic, nutrient-scarce conditions typical of field substation environments. This design was deliberately chosen as an accelerated bio-aging approach. In real-world substations, fungal colonization and subsequent material deterioration occur incrementally over decades. Utilizing a nutrient-rich substrate allows for the rapid establishment of a functional fungal biomass equivalent to long-term environmental exposure within a practical experimental timeframe. Crucially, by comparing the inoculated group with the sterile medium control, the specific contributions of *C. lunata* were clearly decoupled from substrate-induced baseline effects. Although the sterile medium alone caused minor moisture absorption (reaching ~0.8%), the presence of active fungal growth induced significantly greater structural breakdown (e.g., pit formation), chemical alteration (dechlorination and oxidation), and electrical performance degradation. Nevertheless, future studies employing oligotrophic media or other simulated field-like conditions will be valuable to evaluate fungal erosion rates under environments that more closely reflect actual operational scenarios.

From a methodological perspective, establishing a more comprehensive control architecture remains an important goal for future long-term bio-degradation studies. First, incorporating uninoculated sterile medium controls over extended periods will be essential in subsequent research to rule out any subtle baseline material aging induced by the culture substrate itself. Second, while *Curvularia lunata* was targeted here due to its metagenomically verified ecological dominance—a prerequisite for any strain to pose a practical threat in substation environments—introducing non-degrading fungal controls in future work will further validate whether the observed oxidation and dechlorination are uniquely specific to *C. lunata* or represent a generic fungal response. Third, to achieve a more nuanced understanding of the erosion characteristics, heat-killed fungal biomass controls should be employed. Although our FTIR and XPS analyses definitively demonstrate active biochemical degradation, the precise contribution of physical damage (e.g., mechanical hyphal penetration) to the overall structural impairment remains to be quantitatively decoupled from chemical erosion. Addressing these control dimensions in future investigations will provide a more fine-grained mechanistic dissection of polymer bio-deterioration in power grid infrastructure.

In essence, a comprehensive elucidation of the erosion mechanism of *Curvularia lunata* on secondary cable insulation materials was achieved through a systematic application of multidimensional characterization techniques. Microbial colonization and growth were found to induce physical structural deterioration (observed via SEM), heightened hydrophilicity (measured by WCA), elevated water content (quantified using a moisture meter), altered chemical composition (analyzed through FTIR and XPS), and consequent degradation in electrical performance (evaluated by LCR bridge analysis). These outcomes not only enhance our comprehension of microbial corrosion processes but also establish a crucial theoretical foundation for the future development of targeted strategies for safeguarding cables.

## Conclusion

In this study, a systematic investigation was conducted to elucidate the mechanisms of microbial erosion on secondary cable insulation. Metagenomic analysis revealed that the microbial community on contaminated substation cables is dominated by Ascomycota, with *Curvularia lunata* identified as a key fungal species enriched in high-voltage environments.

Through artificial inoculation experiments using the isolated strain *Curvularia lunata B3*, we demonstrated that this fungus drives material degradation through a synergistic physio-chemical process. Physically, the fungal hyphae penetrate the cable surface, creating extensive micro-defects (pores and gullies) and significantly increasing surface roughness. Chemically, enzymatic activity induces the dechlorination and oxidation of the PVC matrix, evidenced by the loss of C-Cl bonds and the formation of polar oxygen-containing functional groups. These structural and chemical alterations result in a marked increase in surface hydrophilicity and moisture retention capacity. Consequently, the electrical performance of the cable is severely compromised, characterized by decreased series resistance and increased capacitance.

Collectively, this work bridges the gap between microbial community ecology and electrical material engineering. It provides compelling evidence that *Curvularia lunata* is a critical agent of insulation failure and establishes a theoretical foundation for developing targeted antifungal strategies and maintenance protocols to ensure the reliability of power systems.
